# Is a Benign Disease Course Possible in Untreated AQP4‐IgG NMOSD?

**DOI:** 10.1111/ene.70049

**Published:** 2025-03-14

**Authors:** Pakeeran Siriratnam, Chiara Rocchi, Emily Gibbons, Patricia Kelly, Samantha Linaker, Saif Huda

**Affiliations:** ^1^ School of Translational Medicine Monash University Melbourne Australia; ^2^ Neurology Department Walton Centre NHS Foundation Trust Liverpool UK; ^3^ Department of Neurology Alfred Health Melbourne Australia

**Keywords:** AQP4, benign, disability, EDSS, NMOSD

## Abstract

**Background:**

Most patients with aquaporin‐4 antibody‐positive neuromyelitis optica spectrum disorder (AQP4‐IgG NMOSD) require life‐long immunosuppression to prevent relapses. Patients who are untreated or undergo de‐escalation of therapy typically experience severe disabling relapses. We present a series of patients who, despite not receiving immunosuppression, developed minimal disability.

**Methods:**

Case series from a UK national NMOSD referral centre. We defined benign disease as an estimated disability status scale score of ≤ 3 after a minimum of 4 years without immunotherapy.

**Results:**

Of 153 AQP4‐IgG NMOSD patients, 8 (5.2%) had a benign disease course after a median follow‐up of 7.5 years (Q1: 5.8, Q3: 13.3) without immunotherapy. All patients were female, and 7/8 were of White racial background. Clinical attacks included isolated optic neuritis, transverse myelitis, area postrema syndrome or combinations of these syndromes.

**Conclusion:**

The presence of benign NMOSD and the potential for safe de‐escalation of therapy in NMOSD remains unclear. This study suggests that both may be possible. Further studies of similar cases could provide valuable insights and identify biomarkers for safe treatment discontinuation.

## 1 Introduction

Neuromyelitis optica spectrum disorder (NMOSD) is a rare autoimmune astrocytopathy associated with aquaporin‐4 antibodies (AQP4‐IgG), characterised by a highly relapsing and disabling disease course [1, 2]. The primary goal of management is to prevent relapses with immunotherapy [3]. The concept of benign NMOSD is controversial and difficult to study due to the disabling nature of the disease [4]. Reluctance to de‐escalate immunotherapy arises from uncertainty about whether severe disability is inevitable for all NMOSD patients [4]. Prior studies of benign NMOSD have focussed on patients receiving continuous immunosuppression [5]. In studies where treatment has been withdrawn, relapses with significant disability have been reported [6].

Identifying the existence of patients who may follow a benign course, even temporarily, is important. This could enable transient suspension of immunosuppression, reducing long‐term side effects. Furthermore, such patients could inform biological and prognostic differences from the commonly observed severe NMOSD phenotype we have come to expect.

## 2 Methods

### 2.1 Study Design and Population

Patients with AQP4‐IgG NMOSD seen between January 2008 and June 2024 at a UK national referral centre were analysed. A benign disease course was defined as an estimated disability status scale (EDSS) score of ≤ 3 without immunosuppressive therapy for at least 4 years following the first clinical presentation of NMOSD, regardless of whether relapses were observed during this time period. Relapses were defined as new symptoms lasting at least 24 h without concurrent infection occurring > 1 month after prior relapse as determined by the treating neurologist [7]. The EDSS was assessed at nadir and recovery 3–12 months later and/or before the next relapse based on either the reported EDSS at the time or retrospectively based on the clinical examination findings. AQP4‐IgG serostatus was confirmed with live cell‐based assay and all patients fulfilled 2015 diagnostic criteria for AQP4‐IgG NMOSD [1].

### 2.2 Ethics Approval and Patient Consent

The study was approved by the Research Ethics Service, NRES Committee London—Hampstead (reference no. 15/LO/1433). All patients provided written informed consent.

### 2.3 Statistical Analysis

Demographic and baseline data are presented as medians and quartiles for continuous variables and as numbers and percentages for categorical variables.

## 3 Results

Of 153 AQP4‐IgG NMOSD patients evaluated, 8 (5.2%) patients had a benign disease course (Table 1). In our cohort, none of the patients who remained without immunotherapy for a minimum of 4 years had a non‐benign disease course. The median age at disease onset was 41 (Q1: 27.8, Q3: 48.2) years. All patients were female (White = 7 and Black = 1). The median disease duration and duration without immunotherapy were 12.6 years (Q1: 10.2, Q3: 17.3) and 7.5 years (Q1: 5.8, Q3: 13.3), respectively. Of onset attacks, five were optic neuritis (ON), two were transverse myelitis (TM) and one was TM and area postrema syndrome. Most patients (6/8; 75%) had a relapsing disease course. AQP4‐IgG serostatus remained positive in 6 of 8 (75%) patients. Apart from the two patients with a monophasic disease course (patients 4 and 5), who had low positive AQP4‐IgG serostatus, all other patients tested clear positive for AQP4‐IgG at least once.

**TABLE 1:** Demographic and clinical features of patients.

	Onset age	Race	Disease duration/yrs	Duration untreated/yrs	Comorbid AUIM	Attack type	Prior therapy	Current therapy	Reason for stopping therapy	No. of attacks	No of attacks off therapy	AQP4‐IgG at attack	Final AQP4‐IgG	Last EDSS	MRI evolution
1	20s	White	17.9	5.5	Nil	TM, TM, TM	Aza, MMF	RTX	Side effects	3	3	+ NT NT	+	3	Subtle changes from C2‐T1 from previous attack but less prominent, MRI brain normal.
2	40s	Black	7.9	7.4	Nil	L ON, L ON	MMF	Nil	Side effects	2	2	NT+	+	3	Previous left optic nerve enhancement, subsequent left optic nerve atrophy. MRI brain and cord normal.
3	50s	White	12.5	7.5	Coeliac disease, dermatitis herpetiform	TM, LETM	Pred, Aza	Nil	Patient choice	2	2	NT +	+	1	Improvement in the thoracic cord lesions, however persistent signal change. MRI brain normal.
4	30s	White	8.5	4.1	Nil	L ON	Aza	Nil	Patient choice	1	1	+	—	2	Left optic nerve atrophy and ongoing signal change. MRI brain and cord normal
5	40s	White	11.8	11.8	Nil	L ON	Nil	Nil	Patient choice	1	1	+	—	0	Resolution of previous left optic radiation signal change, left optic nerve atrophy. MRI cord normal.
6	40s	White	16.6	14.8	Nil	APS/TM, bON/TM, L ON R ON L ON LETM	MMF, Pred	RTX, Pred	Side effects	6	5	NT + NT NT NT +	+	1	Complete resolution of thoracic cord signal change (previous T1–T10 lesion).
7	30s	White	26.1	26.1	Nil	R ON, L ON	Nil	Nil	Patient choice	2	2	NT +	+	2	Follow‐up imaging NA, original MRI showed right optic nerve enhancement
8	20s	White	12.3	6.5	Nil	L ON, L ON, R ON, L ON, L ON, bON, R ON	AZA Pred RTX	RTX	Pregnancy, Patient choice	7	6	NT NT + + NT NT +	+	1	No follow‐up MRI orbits available. MRI brain normal.

Abbreviations: +, positive; APS, area postrema syndrome, Pred, prednisolone; AQP4‐IgG, Aquaporin‐4 antibodies; AZA, azathioprine; LETM, longitudinally extensive TM; MMF, mycophenolate mofetil; NA, not available; NT, not tested; ON, optic neuritis, bilateral (b) left (L) or right (R); RTX, rituximab; TM, transverse myelitis.

**FIGURE 1:**
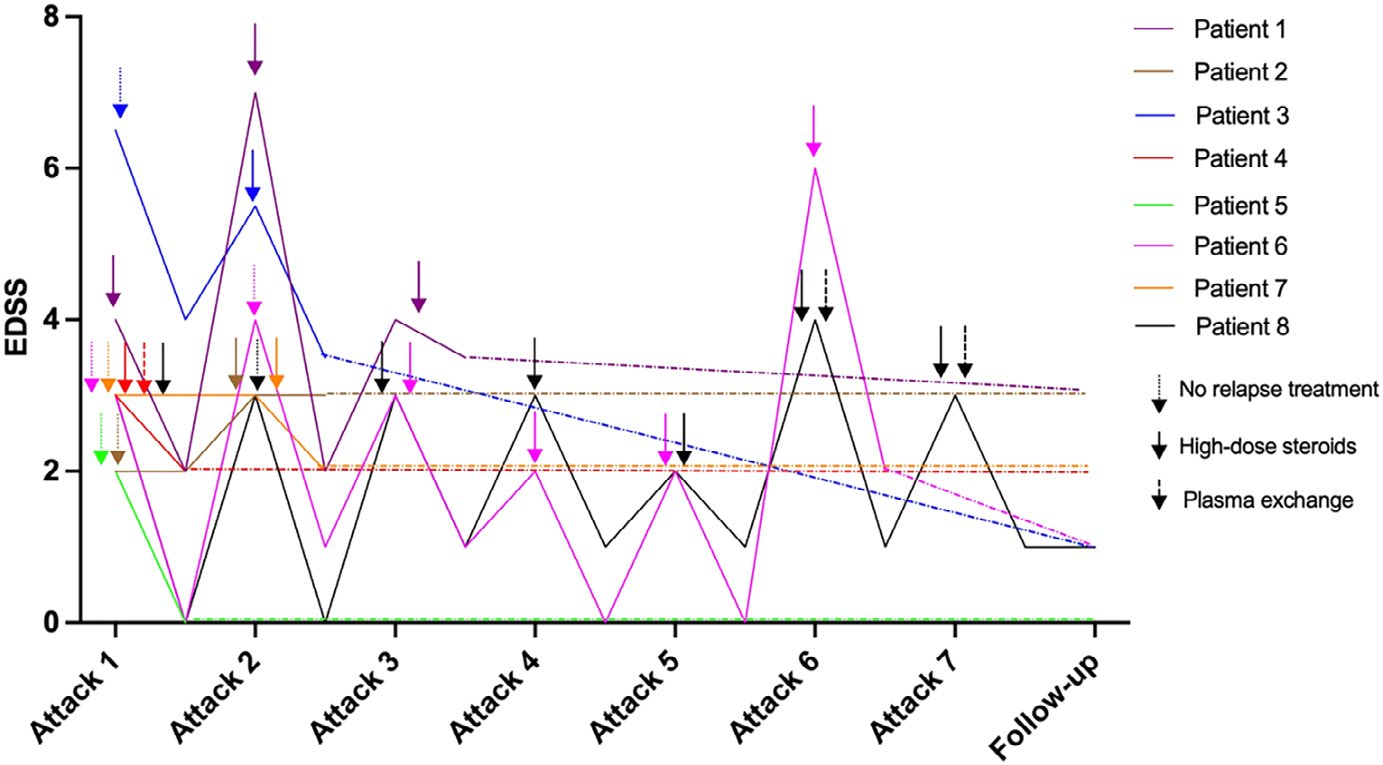
Disability score changes following acute relapses and treatments. Continuous lines represent EDSS changes with each attack (at nadir and recovery). Dashed lines represent attack‐free follow‐up. The colour of treatment symbols and corresponding lines are unique to each patient. Vertical lines during the patient course represent treatment discontinuation points. Patient 6 had a relapse on azathioprine and patient 8 had a relapse on Rituximab. All other relapses occurred off long‐term immunotherapy. EDSS, estimated disability status scale.

There were 22 relapses off long‐term immunotherapy, associated with a relatively mild nadir EDSS of 3 (Q1: 3, Q3: 4) and recovery EDSS of 2 (Q1: 1, Q3: 2). Two relapses occurred on chronic immunotherapy, with a median nadir and recovery EDSS of 3 and 0.5, respectively. However, both of these patients had a total of more than 4 years without immunotherapy, in line with our inclusion criteria. Patients 5 and 7 did not have preventive immunotherapy throughout their disease course. Three patients had more than two relapses without preventive treatment (Patient 1–3 attacks, Patient 6–6 attacks, Patient 8–7 attacks), including longitudinally extensive TM and bilateral ON relapses. At last follow‐up, a favourable median EDSS of 1.5 (Q1: 1, Q3: 2.5) was observed (Figure 1).

Acute treatment was administered in 17/24 (71%) relapses (high‐dose steroids = 17 [3 with plasma exchange]), while the remaining seven attacks were untreated. The median EDSS at nadir and recovery for acutely treated and untreated relapses were similar: 3 (Q1: 3, Q3: 4) and 3 (Q1: 2, Q3: 4) at nadir and 1 (Q1: 1, Q3: 2) and 1 (Q1: 0, Q3: 3) at recovery. Of the seven untreated attacks, two resulted in minimal or no recovery (patient 2's first attack and patient 7's first attack), and one resulted in mild or incomplete recovery (patient 3's first attack). Among the four untreated attacks with complete recovery, three had a nadir EDSS of < 3 (patient 5's only attack, patient 6's first attack and patient 8's second attack). The remaining untreated attacks with complete recovery (patient 6's second attack) had a nadir EDSS of 4. Four of the seven untreated attacks were index presentations, where neuroinflammatory aetiology was not initially suspected. Two attacks (patient 6's first and second attacks) were mild, with rapid spontaneous recovery. Patient 8's second attack was not treated with acute therapy for reasons that remain unclear.

## 4 Discussion

This study identified 5% of AQP4‐IgG NMOSD patients with a benign disease course despite lack of immunosuppression for at least 4 years. Furthermore, most patients had a relapsing disease course but only minimal disability after 7.5 years despite no acute treatment in approximately one‐third of relapses. These cases deviate from the universal expectation of poor outcomes in untreated NMOSD patients and suggest the existence of a potentially less aggressive phenotype in a small subset of patients.

The term ‘benign NMOSD’ is contentious, primarily due to the risk of confusing prolonged remission for a benign course when it may be temporary [4]. Historical studies highlight a 50% risk of blindness and wheelchair use and a 33% risk of death within 5 years of presentation [8]. Consequently, current guidelines advocate lifelong immunosuppression in all cases [1, 2]. With conventional immunosuppression, prior studies have suggested benign AQP4‐IgG NMOSD is rare (4.1% in one study) [5]. Predictors of poor outcomes include Black racial background, older onset age, myelitis and annualised relapse rate > 0.4 [5]. Contemporary monoclonal antibody therapies are likely to address these factors. However, in this study, we were specifically interested in patients with extended treatment‐free periods. Two patients exhibited a monophasic disease course, while three others experienced only two relapses despite being off immunotherapy for at least 4 years. This contrasts with natural history studies of NMOSD, which report significant morbidity and mortality in untreated patients [2]. Remarkably, some patients, including those with myelitis and multiple relapses, also had a benign disease course. This included one patient with two relapses untreated for 26 years and another with six relapses over 17 years (final EDSS 1). However, it is important to acknowledge that 14/17 AQP4‐IgG NMOSD patients in a Korean study relapsed within 6 months of treatment discontinuation, despite > 3 years of prior relapse‐freedom. Three of these relapses led to severe disability despite steroids and plasma exchange [6]. Similarly, two cases of prolonged spontaneous remission (19 and 23 years) eventually resulted in severe visual disability [9]. Although none of the patients in our cohort who remained without immunotherapy for a minimum of 4 years had a non‐benign disease course, this likely reflects selection and confirmation bias as patients with more severe relapses while without immunotherapy for shorter periods may be more likely to consent to immunotherapy.

While relapse prevention remains central to NMOSD management, there is increasing recognition of the potential complications of long‐term immunotherapy. A recent study found that nearly half of NMOSD mortality was unrelated to the disease itself, with a further 10.3% of deaths attributable to immunotherapy complications [10]. A major challenge in de‐escalating immunotherapy is the lack of biomarkers to guide treatment suspension or cessation. Although some studies have suggested negative AQP4‐IgG seroconversion is associated with reduced relapse risk, [11] and our two patients with a monophasic disease course did experience negative seroconversion, this does not appear to be a consistently reliable biomarker for predicting relapse risk [12, 13].

An additional important observation was the recovery from attacks despite lack of acute relapse management. One‐third of the attacks were untreated, including one instance where the patient had an EDSS of 6.5. However, physical therapy may have accounted for some of the improvements. Previous studies have demonstrated that the absence or delay in acute relapse treatment is associated with limited recovery, relapse‐associated worsening and increased disability accrual [3, 14, 15]. In addition to the clinical improvement, MRI lesion resolution was seen in many cases, which is atypical in NMOSD [2]. The spontaneous recovery observed in our patients is likely attributable to a less aggressive NMOSD phenotype, although the exact reasons remain unclear. EDSS was calculated retrospectively from clinical notes in some patients, and not all attacks were investigated with MRI or optical coherence tomography. Nevertheless, our findings suggest that relapsing NMOSD may present along a spectrum of severity underscoring the importance of individualising treatment decisions.

This case series highlights a subgroup of NMOSD patients with a benign disease course despite relapses and not receiving immunotherapy. Highlighting the existence of such cases is of importance as it challenges the notion of universally poor outcomes in NMOSD without treatment. Moreover, these cases may inform biomarkers associated with possible immune tolerance and favourable prognosis. While the prospect of permanent immunosuppression discontinuation currently remains out of reach, exploring the efficacy and safety of periodic immunotherapy de‐escalation protocols incorporating clinical and serological (e.g., glial fibrillary acidic protein) markers could help mitigate immunosuppression‐associated risks and individualise long‐term NMOSD management.

## Author Contributions

**Pakeeran Siriratnam:** conceptualization, methodology, investigation, validation, formal analysis, writing – original draft, writing – review and editing. **Chiara Rocchi:** investigation, validation, writing – review and editing. **Emily Gibbons:** investigation, validation, writing – review and editing. **Patricia Kelly:** investigation, validation, writing – review and editing. **Samantha Linaker:** investigation, validation, writing – review and editing. **Saif Huda:** conceptualization, methodology, investigation, validation, supervision, formal analysis, writing – original draft, writing – review and editing.

## Conflicts of Interest

P.S. has received travel support from Novartis and Biogen; S.H. is partly funded by the National Institute for Health Research SCPRA grant and NHS England Highly Specialised Services. The remaining authors declare no conflicts of interest with respect to the research, authorship and/or publication of this article.

## Data Availability

The data that support the findings of this study are available on request from the corresponding author. The data are not publicly available due to privacy or ethical restrictions.
